# Evogliptin Directly Inhibits Inflammatory and Fibrotic Signaling in Isolated Liver Cells

**DOI:** 10.3390/ijms231911636

**Published:** 2022-10-01

**Authors:** Hye-Young Seo, So-Hee Lee, Eugene Han, Jae Seok Hwang, Sol Han, Mi Kyung Kim, Byoung Kuk Jang

**Affiliations:** 1 Department of Internal Medicine, School of Medicine, Institute for Medical Science, Keimyung University, Daegu 42601, Korea; 2 Department of Physiology, University of Washington, Seattle, WA 98195, USA

**Keywords:** evogliptin, Kupffer cells, hepatocytes, hepatic stellate cells, LPS, TGF-β, autophagy

## Abstract

Chronic liver inflammation can lead to fibrosis, cirrhosis, and hepatocellular carcinoma. Kupffer cells (KC) secrete proinflammatory and fibrogenic cytokines in response to lipopolysaccharide (LPS), and so play an important role in liver inflammation, where they induce hepatocellular damage. LPS also activates hepatic stellate cells and induces extracellular matrix deposition. In this study, we used isolated primary KC, primary hepatocytes, and primary hepatic stellate cells (HSC) to investigate whether evogliptin directly inhibits inflammatory and fibrotic signaling. We found that evogliptin inhibited LPS-induced secretion of inducible nitric oxide synthase and transforming growth factor β (TGF-β) from KC. Moreover, evogliptin inhibited inflammatory mediator release from hepatocytes and hepatic stellate cell activation that were induced by KC-secreted cytokines. In hepatocytes, evogliptin also inhibited LPS-induced expression of proinflammatory cytokines and fibrotic TGF-β. In addition, evogliptin inhibited TGF-β-induced increases in connective tissue growth factor levels and HSC activation. These findings indicate that evogliptin inhibits inflammatory and fibrotic signaling in liver cells. We also showed that the inhibitory effect of evogliptin on inflammatory and fibrotic signaling is associated with the induction of autophagy.

## 1. Introduction

Inflammation occurs during all stages of liver disease, yet chronic liver inflammation that leads to fibrosis, cirrhosis, and hepatocellular carcinoma is a particular health concern [1]. Kupffer cells (KC), which play an important role in liver inflammation, are resident macrophages that make up approximately 30% of sinusoidal cells [2]. KC are activated in response to liver injury when exposed to substances such as lipopolysaccharide (LPS), a product of intestinal bacteria. KC also produce various inflammatory cytokines, such as interleukin (IL)1α, IL1β, tumor necrosis factor (TNF)α, and IL-6, and release reactive oxygen species (ROS) to increase levels of inducible nitric oxide synthase (iNOS) [3,4,5]. Furthermore, activated KC and damaged hepatocytes promote the secretion of proinflammatory and fibrogenic cytokines from macrophages, thereby activating hepatic stellate cells (HSC) and inducing extracellular matrix deposition [6,7]. The secretion of these inflammatory cytokines induces liver damage, which is a major cause of chronic liver inflammation and fibrosis [8,9]. As such, inflammation and fibrosis are mediated by the interactions between KC, hepatocytes, and HSC. Connective tissue growth factor (CTGF) is a major profibrogenic cytokine that is transcriptionally activated by TGF-β [10]. Increased levels of CTGF stimulate the synthesis of collagen and fibronectin, which leads to TGF-β-induced fibrosis [11,12].

Dipeptidyl peptidase-4 (DPP4, also known as CD26) is a serine peptidase that is ubiquitously expressed in various organs [13,14]. DPP4 inhibitors maintain glucose homeostasis by preventing the degradation of glucagon-like peptide 1 (GLP-1), and also act on additional targets including the peptides stromal cell-derived factor-1 and neuropeptide Y. In addition, DPP4 is an adipokine that is involved in immune-cell activation [15]. Based on these various effects of DPP4 inhibitors, previous studies have investigated the anti-inflammatory and antifibrotic effects of DPP4 inhibitors in various organ systems using animal models [15,16,17,18].

Recent studies show that DPP4 expression is elevated in the liver of patients with nonalcoholic fatty liver disease (NAFLD) [19]. In addition, several DPP4 inhibitors are effective in limiting steatosis, liver inflammation, and fibrosis in humans [20,21,22,23].

Evogliptin is an effective and safe DPP4 inhibitor approved for treating type 2 diabetes mellitus (T2DM) [24], Although a recent study showed that evogliptin reduces the hepatic fatty burden in patients with T2DM and NAFLD [25], studies on the direct effect of evogliptin on liver inflammation and fibrosis are limited. In this study, we investigated the effect of evogliptin on the release and expression of inflammatory and fibrotic mediators from primary KC, primary hepatocytes, and primary HSC, to determine if evogliptin might directly protect against liver inflammation and fibrosis.

## 2. Results

### 2.1. Evogliptin Inhibited Cytokine Secretion from Kupffer Cells

**Figure 1:**
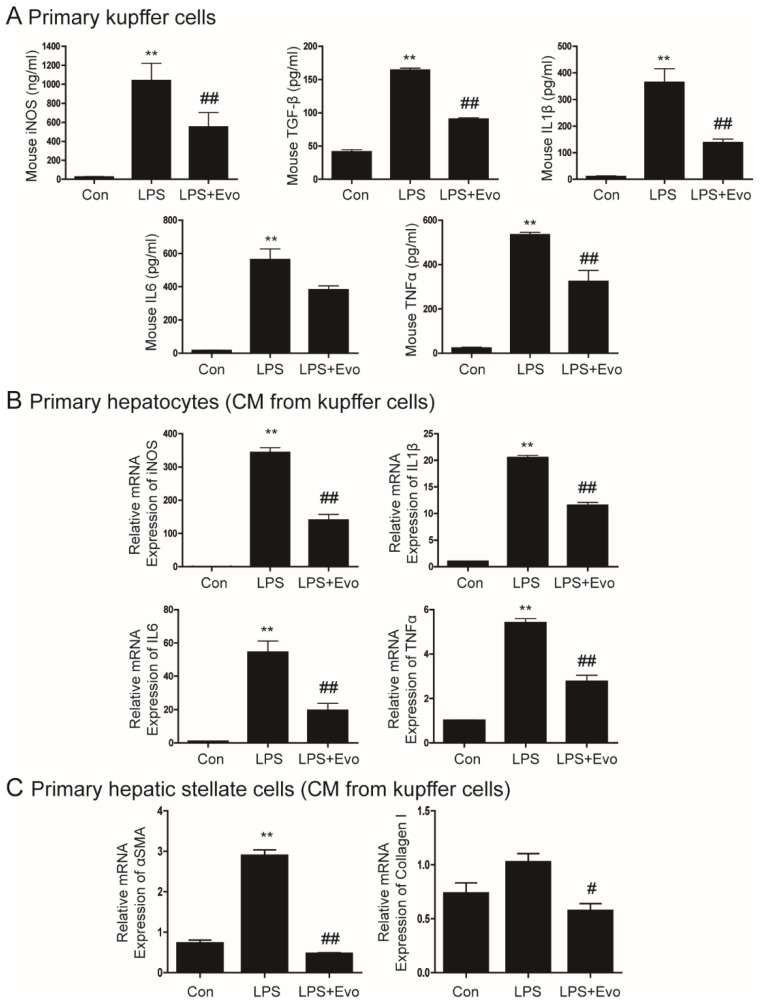
The inhibitory effect of evogliptin on lipopolysaccharide (LPS)-induced cytokine secretion from Kupffer cells. (**A**) Primary Kupffer cells (KC) were pretreated with LPS for 2 h and then treated with evogliptin. After 24 h, the media was collected and measured using ELISA. The data are the mean ± SEM of three independent measurements. ** *p* < 0.01 relative to the control, ^##^ *p* < 0.01 relative to LPS. (**B**) Representative real-time RT-PCR analysis of the expression levels of iNOS, IL1β, IL6, and TNFα in primary hepatocytes treated with conditioned media (CM) from LPS-treated primary KC. The data are the mean ± SEM. ** *p* < 0.01 relative to the control, ^##^ *p* < 0.01 relative to LPS. (**C**) Representative real-time RT-PCR analysis of the expression levels of αSMA and collagen in primary hepatocytes treated with CM from LPS-treated primary hepatic stellate cells. Data are the mean ± SEM. ** *p* < 0.01 relative to the control, ^#^ *p* < 0.05, ^##^ *p* < 0.01 relative to LPS.

LPS is a proinflammatory molecule involved in liver damage, inflammation, and fibrosis [4]. First, we evaluated whether evogliptin affects liver inflammation and fibrosis. KC were treated with LPS and evogliptin, and then the release of inflammatory cytokines and the profibrogenic cytokine TGF-β was measured. As expected, LPS significantly increased the secretion of iNOS, TGF-β, IL1β, IL6, and TNFα (Figure 1A). These cytokines were significantly decreased after evogliptin treatment (Figure 1A).

### 2.2. Evogliptin Inhibited Hepatocyte Inflammation and Hepatic Stellate Cell Activation Caused by KC-Secreted Cytokines

Activated KC secrete cytokines, which further increases hepatocyte inflammation [6,9]. We next investigated whether evogliptin inhibits inflammation induced by KC-secreted cytokines. The mRNA levels of iNOS, IL1β, IL6, and TNFα were higher in hepatocytes incubated in CM obtained from LPS-treated KC than controls, and were significantly inhibited by evogliptin. (Figure 1B). Crosstalk between KC and HSC is important for the activation of HSC and initiation of liver fibrosis [6,9]. Therefore, we treated primary HSC with CM from LPS-treated KC. Our results showed that αSMA and collagen levels were higher in the CM, and that evogliptin treatment decreased αSMA and collagen levels (Figure 1C).

### 2.3. Evogliptin Inhibited LPS-Stimulated Inflammatory Cytokine Levels in Primary Hepatocytes

**Figure 2:**
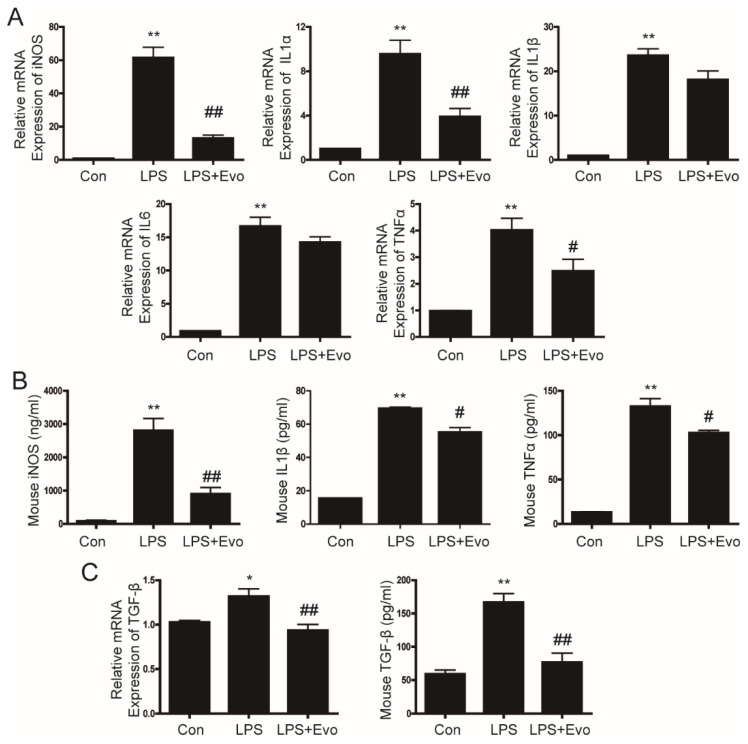
The inhibitory effect of evogliptin on LPS-induced cytokine levels in hepatocytes. Primary hepatocytes were pretreated with LPS for 2 h and then treated with evogliptin for 24 h. (**A**) Representative real-time RT-PCR analysis of the expression levels of iNOS, IL1α, IL1β, IL6, and TNFα. Data are the mean ± SEM. ** *p* < 0.01 relative to the control, ^##^ *p* < 0.01 relative to the LPS. (**B**) The production of iNOS and the secretion of IL1β and TNFα were measured using ELISA. Data are the mean ± SEM. ** *p* < 0.01 relative to the control, ^#^ *p* < 0.05, ^##^ *p* < 0.01 relative to LPS. (**C**) TGF-β mRNA was measured by real-time RT-PCR, and TGF-β secretion was measured by ELISA. Data are the mean ± SEM. * *p* < 0.05, ** *p* < 0.01 relative to the control, ^##^ *p* < 0.01 relative to LPS.

Next, we examined the effects of evogliptin on the levels of inflammatory cytokines in primary hepatocytes. LPS increased the iNOS, IL1α, IL1β, IL6, and TNFα mRNA expression and secretion of inflammatory cytokines (Figure 2A,B). After evogliptin treatment, iNOS mRNA and production were significantly decreased, as were the iNOS, IL1α, and TNFα mRNA expression and iNOS, IL1β and TNFα secretion (Figure 2A,B). In addition, TGF-β mRNA and secretion were increased by LPS and inhibited by evogliptin treatment (Figure 2C). These results indicate that evogliptin inhibits the secretion and expression of LPS-induced proinflammatory cytokines and the profibrogenic cytokine TGF-β in primary hepatocytes.

### 2.4. Evogliptin Inhibited TGF-β-Induced CTGF Expression and Smad3 Phosphorylation

**Figure 3:**
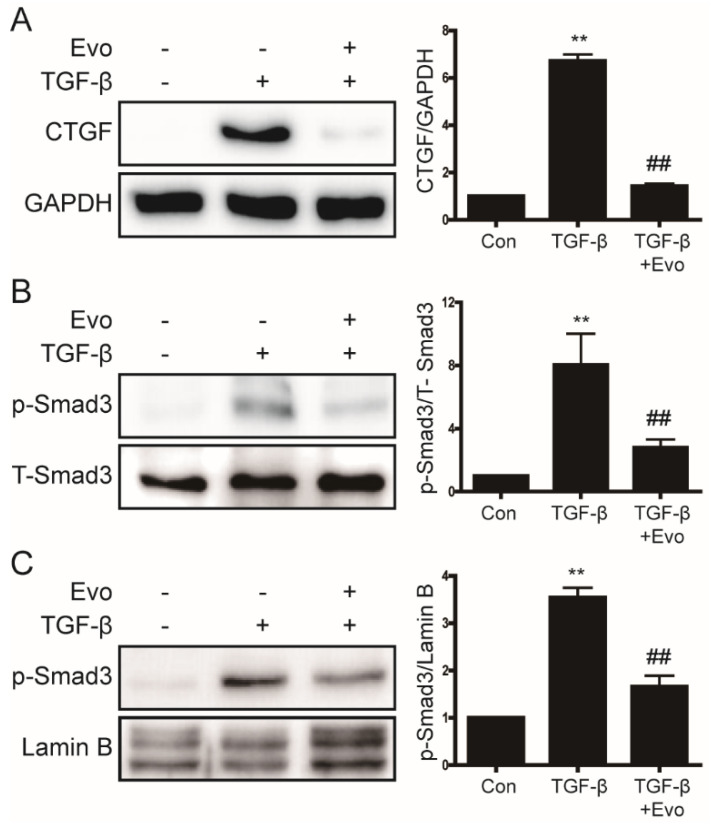
The inhibitory effect of evogliptin on TGF-β-induced CTGF levels and p-Smad3 expression. (**A**,**B**) Western blot analysis of the effects of evogliptin on TGF-β-induced CTGF levels and p-Smad3 expression in primary hepatocytes. Data in the bar graphs are represented as the mean ± SEM. ** *p* < 0.01 relative to the control, ^##^ *p* < 0.01 relative to TGF-β. (**C**) Western blot analyses of nuclear extracts from primary hepatocytes showing the effect of evogliptin on TGF-β-induced p-Smad3 expression. ** *p* < 0.01 relative to the control, ^##^ *p* < 0.01 relative to TGF-β.

LPS-mediated increases in TGF-β levels induce liver fibrosis and increase the expression of CTGF [12,26]. Therefore, we investigated the effects of evogliptin on TGF-β-induced CTGF expression and Smad3 phosphorylation in primary hepatocytes. As shown in Figure 3A, evogliptin attenuated TGF-β-induced CTGF expression. Upon TGF stimulation, Smad3 is activated by phosphorylation and translocate to the nucleus, where it activates a fibrotic signaling pathway that leads to liver fibrosis [27]. Next, we investigated whether evogliptin inhibits Smad3 phosphorylation. As shown in Figure 3B,C, evogliptin inhibited TGF-β-induced Smad3 phosphorylation and nuclear translocation. These results suggest that by attenuating the phosphorylation of Smad3, evogliptin inhibits a pathway that is involved in liver fibrosis.

### 2.5. Evogliptin Increased Autophagy Flux

**Figure 4:**
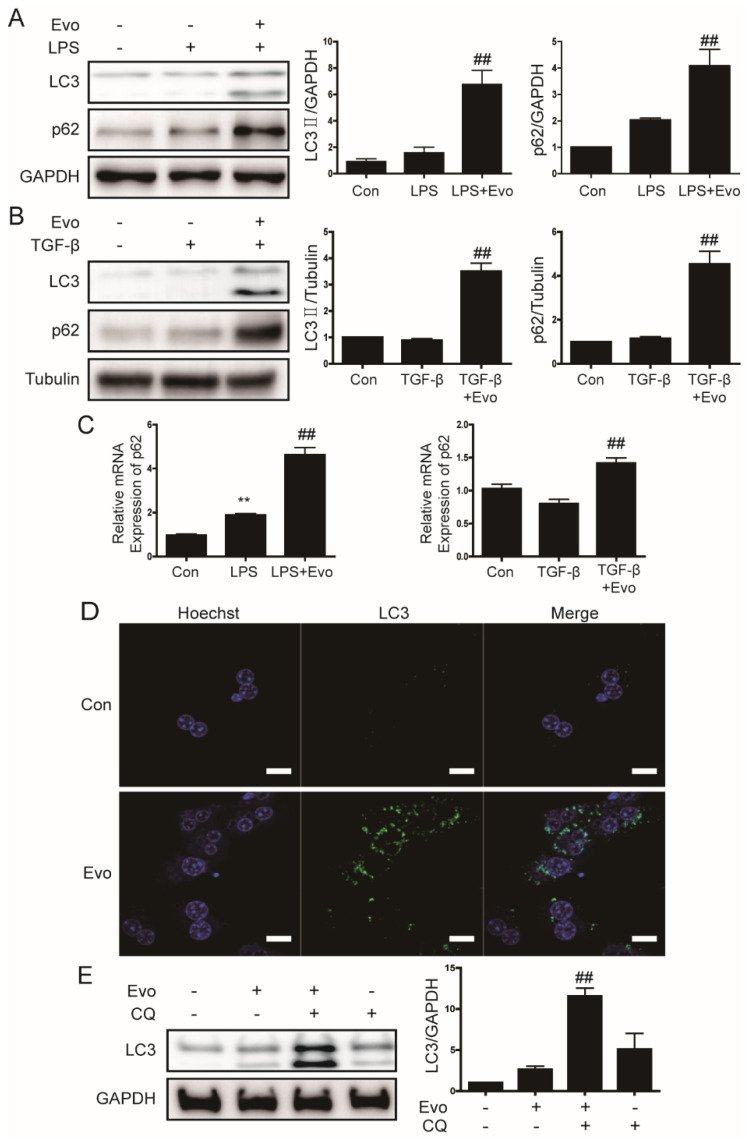
The effect of evogliptin on autophagy. (**A**,**B**) Representative Western blots showing the effects of evogliptin on LC3 and p62 levels in primary hepatocytes. Data in the bar graphs are represented as the mean ± SEM. ^##^ *p* < 0.01 relative to LPS or TGF-β. (**C**) Representative real-time RT-PCR analysis of the effect of evogliptin on p62 mRNA levels. Data in the bar graph are the mean ± SEM. ** *p* < 0.01 relative to the control, ^##^ *p* < 0.01 relative to LPS/TGF-β. (**D**) Primary hepatocytes were treated with evogliptin, and LC3 puncta formation was analyzed by immunofluorescence; original magnification ×800. (**E**) A representative Western blot showing the effect of evogliptin and chloroquine (CQ, 10 μM) on LC3 protein levels in primary hepatocytes. Data in the bar graph are represented as the mean ± SEM. ^##^ *p* < 0.01 relative to the evogliptin or chloroquine.

A recent study reported that the DPP4 inhibitor sitagliptin improves insulin resistance by inhibiting the inflammatory response and activating autophagy [28]. Therefore, we next examined the effect of evogliptin on components of the autophagy pathway in primary hepatocytes. Evogliptin significantly increased the expression of LC3 II protein and LC3 puncta (Figure 4A,B,D). Evogliptin also increased the expression of p62 protein, but this result was likely nonspecific due to a global increase in mRNA levels (Figure 4C). Therefore, we used the autophagy inhibitor chloroquine to determine whether evogliptin increased autophagy flux. The results indicated that LC3 II protein expression was higher when co-treated with evogliptin and chloroquine (Figure 4E); this finding suggests that evogliptin increases autophagy flux.

### 2.6. Inhibition of Inflammatory Signaling by Evogliptin Did Not Require Modulation of the Nrf2/HO-1 Pathway

An increase in p62 levels induces the release of Keap1 (Kelch-like ECH-associated protein) from Nrf2 (nuclear factor erythroid 2-related factor 2), leading to nuclear translocation and activation of Nrf2 [29]. In addition, recent studies have reported that the DPP-4 inhibitors saxagliptin and linagliptin activate the Nrf2/HO-1 pathway [30,31], so we investigated whether evogliptin induces Nrf2 and HO-1 expression. As shown in Figure S1A, evogliptin induced Nrf2 and HO-1 protein expression in primary hepatocytes. To elucidate the role of the Nrf2/HO-1 pathway in the effects of evogliptin on inflammatory signaling, we suppressed the expression of Nrf2 with siRNA-Nrf2 (Figure S1B). However, despite inhibited Nrf2 and HO-1 expression, evogliptin still inhibited the mRNA expression of proinflammatory cytokines (Figure S1C). This result suggests that inhibition of inflammatory signaling by evogliptin does not require modulation of the Nrf2/HO-1 pathway.

### 2.7. Evogliptin Prevents Inflammatory and Fibrotic Signaling through Autophagy Induction

**Figure 5:**
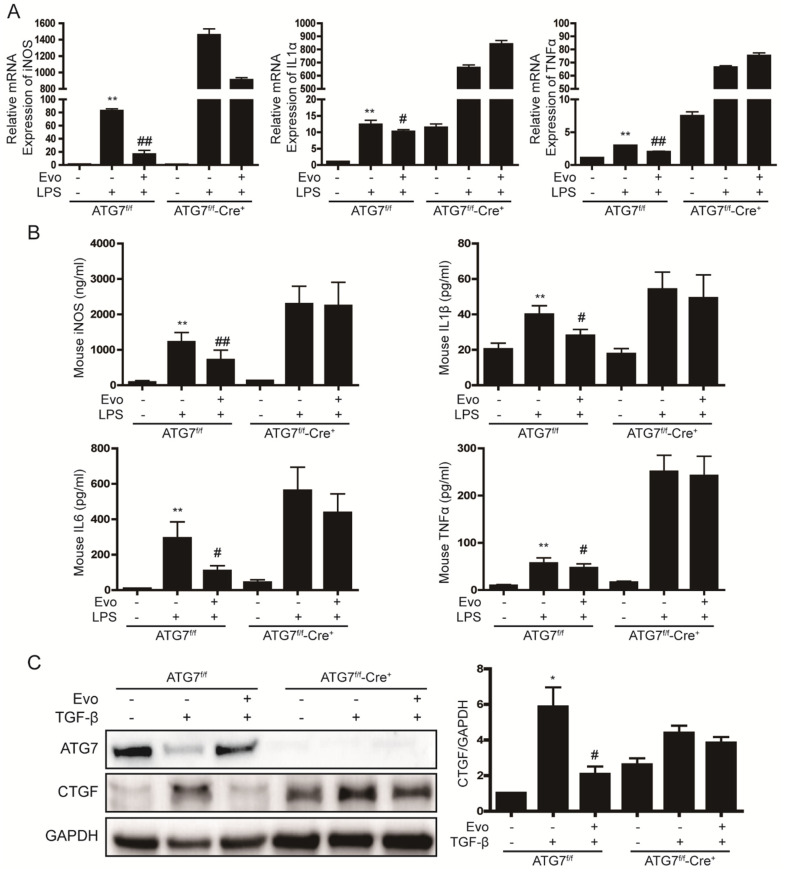
Induction of autophagy is required for evogliptin to prevent inflammatory and fibrotic signaling in liver cells. (**A**) Real-time RT-PCR analysis of the effect of evogliptin on LPS-induced inflammatory signaling in primary hepatocytes from ATG7^f/f^-Cre^+^ mice. ** *p* < 0.01 relative to the control,^*#*^ *p < 0.05,* ^##^ *p* < 0.01 relative to LPS. (**B**) iNOS production and cytokine secretion in primary hepatocytes from ATG7^f/f^-Cre^+^ mice. ** *p* < 0.01 relative to the control, ^#^ *p* < 0.05, ^##^ *p* < 0.01 relative to LPS. (**C**) Western blot analysis of the effect of evogliptin on TGF-β-induced CTGF expression in primary hepatocytes from ATG7^f/f^-Cre^+^ mice. Data in the bar graph are represented as the mean ± SEM. * *p* < 0.05 relative to the control, ^#^ *p* < 0.05 relative to TGF-β.

Next, we used hepatocyte-specific ATG7 knockout mice to investigate the association between autophagy and inflammatory and fibrotic signaling in liver cells. In primary hepatocytes from ATG7^f/f^-Cre^+^ mice, evogliptin showed a tendency to decrease iNOS mRNA levels (Figure 5A) but did not reduce iNOS production or IL1β, IL6, and TNFα secretion (Figure 5B). In addition, evogliptin did not decrease CTGF expression in primary hepatocytes from ATG7^f/f^-Cre^+^ mice (Figure 5C). This result suggests that evogliptin decreases inflammatory and fibrotic signaling by inducing autophagy.

## 3. Discussion

In this study, we used primary KC, primary hepatocytes, and primary HSC to show that evogliptin directly inhibited inflammatory and fibrotic signaling in liver cells. Moreover, we found that the inhibitory effect of evogliptin on inflammatory and fibrotic signaling was associated with the induction of autophagy.

LPS is a potent proinflammatory molecule associated with liver injury, inflammation, and fibrosis [32]. KC that are activated by LPS produce large amounts of ROS, proinflammatory cytokines, and chemokines, and induce the infiltration of other inflammatory cells; together, these actions impair the function and viability of adjacent cells [5,33]. In the continued presence of the damaging stimulus, dying hepatocytes, KC, recruited lymphocytes, and HSC contribute to a persistent inflammatory environment [33]. In addition, activated KC activate HSC, leading to extracellular matrix deposition. Several studies revealed the anti-inflammatory and antifibrotic effects of evogliptin [34,35,36,37]; however, few studies have investigated its direct effect on the liver. We hypothesized that evogliptin inhibits fibrotic signaling by inhibiting inflammatory signaling in the liver.

Recent studies have shown that increased DPP4 expression in the liver promotes NAFLD, which is associated with a decrease in active GLP-1 levels and the effect of DPP4 on insulin signaling in mice [19]. In addition, DPP4 can stimulate macrophages to increase inflammatory processes at both systemic and local levels [38]. Therefore, there is a limitation in identifying the GLP-1/DPP4-independent pathway in in vivo model. In this study, we determined the effect of evogliptin in each cell type using primary KC, primary hepatocytes, and primary HSC, which play important roles in liver inflammation and fibrosis. First, we showed that the secretion of proinflammatory cytokines was increased by LPS treatment in KC and inhibited by evogliptin treatment. In addition, when hepatocytes and HSC were treated with cytokines secreted from KC, pro-inflammatory cytokines were increased in hepatocytes and HSCs were activated. Therefore, the effects of LPS not only activates KC but also promotes hepatocyte damage and hepatic stellate cell activation, and these effects synergistically induce liver inflammation and fibrosis. In particular, we showed that evogliptin inhibited LPS-induced iNOS and TGF-β secretion from KC. The onset of chronic liver disease usually involves an inflammatory phase that progresses to fibrosis after sustained oxidative stress, in which iNOS is upregulated [39]. TGF-β represents an important link between immune cells and fibrotic cells [1]. The majority of TGF-β is produced by immune cells, including liver macrophages, to directly promote fibrosis [40]. Therefore, inhibition of iNOS and TGF-β secretion by evogliptin in KC also reduced inflammatory and fibrotic signaling in hepatocytes and HSC. We investigated whether evogliptin is directly involved in HSC activation. At 7 days after the HSC were isolated, the cells were activated, and αSMC levels increased; these effects were suppressed by evogliptin treatment (Figure S2A,B). Evogliptin also inhibited the increased collagen and CTGF expression that was observed during HSC activation (Figure S2C). Our results suggested that the anti-inflammatory and anti-fibrotic properties of evogliptin are pathways independent of GLP-1/DPP4 involved in systemic anti-inflammatory and glucose management.

Autophagy is a lysosomal degradation pathway that maintains cellular homeostasis [41]. In the liver, autophagy protects hepatocytes by removing damaged organelles and proteins. Autophagy plays an important role in inflammation by influencing the development, homeostasis and survival of inflammatory cells such as macrophages, neutrophils and lymphocytes. Therefore, modulation of autophagy may be a mechanism that could be used to treat diseases related to inflammation [42]. Recent studies reported that DPP4 inhibitors increase autophagy [28,43]. In the current study, both LC3 II and p62 levels were increased by evogliptin, and evogliptin consequently increased autophagy flux. An increase in p62 levels is known to induce activation of Nrf2 [29]. In this current study, evogliptin increased the protein expression of Nrf2 and HO1 (a target gene of Nrf2), but the increase in Nrf2 expression did not inhibit inflammatory signaling (Figure S1). On the other hand, evogliptin did not decrease inflammatory cytokine and CTGF expression in primary hepatocytes from ATG7^f/f^-Cre^+^ mice, in which autophagy is completely blocked (Figure 5). The role of autophagy in the liver has been widely reported but remains controversial as both positive and negative effects have been reported, especially in studies of liver fibrosis [44]. In this study, evogliptin reduced liver inflammatory signaling through a pathway that involved autophagy and inhibited liver fibrotic signaling.

In conclusion, our results show that evogliptin decreased LPS-stimulated inflammatory cytokine secretion, TGF-β-induced CTGF expression, and HSC activation. Evogliptin is a DPP4 inhibitor, a class of drugs commonly used to control blood sugar levels in patients with T2DM. Our data suggest that evogliptin may be a beneficial treatment option for the treatment of liver inflammation and fibrosis in diabetic patients.

## 4. Materials and Methods

### 4.1. Chemicals

Lipopolysaccharide (LPS, Escherichia coli 055; B5) was purchased from Sigma-Aldrich (St. Louis, MO, USA). Recombinant human TGF-β (5 ng/mL) was purchased from R&D Systems (Minneapolis, MN, USA). Anti-CTGF (SC365970) and anti-HO-1 (SC10789) antibodies were purchased from Santa Cruz Biotechnology (Dallas, TX, USA), and an anti-p62 (ab56416) antibody was purchased from Abcam (Cambridge, UK). An anti-Nrf2 (PA527882) antibody was purchased from Thermo Fisher Scientific (Waltham, MA, USA). Anti-phospho-Smad3 (Ser423/425) (CS9520), anti-GAPDH (CS2118), antitubulin (CS2146), anti-LC3B (CS2775), and anti-ATG7 (CS2631) antibodies, and antirabbit (7074P2) and antimouse (7076P2) secondary antibodies, were purchased from Cell Signaling Technology (Beverly, MA, USA).

### 4.2. Isolation of Primary KC and Primary Hepatocytes

KC and hepatocytes were obtained by perfusion of EGTA and collagenase through the portal vein of C57BL/6 mice. After shaking for 20 min in a 37 °C incubator, hepatocytes were obtained by filtration through a 70 μm nylon mesh. Centrifuge at 500 rpm for 5 min to separate the hepatocyte pellet and the supernatant with KC. Hepatocytes were resuspended in Williams’ medium E (Sigma-Aldrich), cultured in type I collagen-coated dishes (IWAKI Scitech Kiv, Tokyo, Japan) for 1–2 h, and then cultured with medium 199 (Sigma-Aldrich). The viability of hepatocytes was always higher than 90%. The hepatocyte pellet and the separated supernatant were centrifuged at 1600 rpm for 10 min, and then the pellet was subjected to OptiPrep (Sigma-Aldrich) density-gradient centrifugation to separate KC. The isolated KC was plated on RPMI 1640 medium (Gibco-BRL, Grand Island, NY, USA) containing 10% fetal bovine serum and incubated for 30 min, then the media was replaced to obtain purified KC. The viability of KC was always higher than 90%. Primary cells were pretreated with compounds in 0.5% FBS with or without LPS (1 μg/mL) for 2 h, followed by evogliptin (100 μM) for 24 h. The isolation method previously described was used [45].

### 4.3. Isolation of Primary HSC

HSCs were isolated by perfusing the liver through the inferior vena cava of C57BL/6 mice. Perfusion was performed in the following order; EGTA buffer, pronase (Roche Diagnostics, Indianapolis, IN, USA), and collagenase (Roche Diagnostics). The liver was shaken in an incubator at 37 °C for 20 min, filtered through a 70 μm nylon mesh, and centrifuged at 1625 rpm for 10 min. The pellet of isolated hepatocytes was mixed and gently overlayed with a gradient of OptiPrep (Sigma-Aldrich). The sample was then centrifuged at 3000 rpm for 17 min at 4 °C without braking. HSCs present in a thin white layer at the interface between OptiPrep and HBSS were harvested and washed with HBSS. HSCs were plated in DMEM (Gibco-BRL) containing 10% FBS. The viability of HSC was always higher than 90%. The isolation method previously described was used [45].

### 4.4. Quantitative Real-Time PCR

Total RNA was isolated from cells, using Trizol reagent (Invitrogen, Waltham, MA, USA). cDNA was prepared using a Maxima First Strand cDNA synthesis kit (Thermo Fisher Scientific). Real-time RT-PCR was performed using a SYBR Green PCR master mix kit (Roche Diagnostics, Indianapolis, IN, USA) and a CFX Connect real-time PCR system (Bio-Rad, Richmond, CA, USA). PCR primers were as follows: 45 cycles of 95 °C for 30 s, 60 °C for 10 s, and 72 °C for 15 s. Primer sequences were as follows: mouse iNOS forward, 5′-CATGCTACTGGAGGTGGGTG-3′, and reverse, 5′-CATTGATCTCCGTGACAGCC-3′; mouse TGF-β forward, 5′-AAATCAACGGGATCAGCCCC-3′, and reverse, 5′-GGATCCACTTCCAACCCAGG-3′; mouse p62 forward, 5′-AAGTTCCAGCACAGGCACAG-3′, and reverse, 5′-CTCCTCCTGAGCAGTTTCCC-3′; mouse IL1α, 5′-CAACGTCAAGCAACGGGAAG-3′, and reverse, 5′-AAGGTGCTGATCTGGCTTGG-3′; mouse IL1β forward, 5′-CTTTCCCGTGGACCTTCCAG-3′, and reverse, 5′-AATGGGAACGTCACACACCA-3′; mouse IL6 forward, 5′-TTGCCTTCTTGGGACTGATG-30, and reverse, 5′-CTCATTTCCACGATTTCCCA-3′; mouse TNFα forward, 5′-ACCGTCAGCCGATTTGCTAT-3′, and reverse, 50-CCGGACTCCGCAAAGTCTAA-3′; mouse GAPDH forward, 5′-ACGACCCCTT CATTGACCTC-3′, and reverse, 5′-ATGATGACCCTTTTGGCTCC-3′.

### 4.5. Determination of Cytokine Levels

KC and hepatocytes were seeded in 6-well plates and treated with LPS and evogliptin. Cells and supernatant fractions were harvested and then centrifuged at 1000 rpm for 5 min, and cell pellets or CM were stored at −80 °C. Levels of iNOS (Abcam), TGF-β, IL1α, IL1β, IL6, and TNFα (R&D Systems, Abingdon, UK) were detected using ELISA kits according to the manufacturers’ instructions.

### 4.6. Western Blot Analysis

The cells were incubated with the RIPA buffer (Thermo Fisher Scientific) containing protease/phosphatase inhibitors (an inhibitor cocktail solution; genDEPOT, Katy, TX, USA) for 30 min at 4 °C. Protein concentrations were determined using a BCA assay (Thermo Fisher Scientific). Equal amounts of solubilized proteins were separated using SDS-PAGE and then transferred to PVDF membranes (Millipore, Billerica, MA, USA). The membranes were blocked with 5% skim milk prepared in TBST (Tris-buffered saline containing 0.1% Tween 20), and incubated with primary antibodies and then secondary antibody. Protein bands were detected using enhanced Clarity™ Western ECL substrate kit (Bio-Rad). Signal intensities were quantified using densitometry with ImageJ software version 1.52a (NIH, Bethesda, MD, USA).

### 4.7. ATG7^f/f^-Albumin-Cre^+^ Mice

ATG7 floxed mice (ATG7^f/f^) were bred with albumin-Cre mice to generate ATG7 hepatocyte-specific knockout mice (ATG7^f/f^ Alb-Cre^+^). The mice were a kind gift from Dr. Myung-Shik Lee (Yonsei University) with the permission of Dr. Masaaki Komatsu (Tokyo Metropolitan Institute of Medical Science). All experiments were approved by the Institutional Animal Care and Use Committee of Keimyung University (KM-2019-14R3). Genotyping of the offspring was performed by PCR as previously described [46].

### 4.8. Immunofluorescence Analysis

Primary hepatocytes were fixed in 10% formalin and then permeabilized with 0.1% Triton-X100 for 15 min. After incubation with anti-LC3 and Alexa Fluor-conjugated secondary antibodies (Abcam), hepatocytes were imaged using a confocal microscope (Carl Zeiss, Oberkochen, Germany).

### 4.9. siRNA Targeting Nrf2

Predesigned siRNA for NRF2 were purchased from Santa Cruz Biotechnology. Cells were transfected with 50 nM siRNA using the Lipofectamine RNAiMAX reagent (Invitrogen, Carlsbad, CA, USA) according to the manufacturer‘s instructions. The effects of Nrf2 siRNA on the expression of endogenous NRF2 were confirmed by real-time RT-PCR or Western blot analysis.

### 4.10. Statistical Analysis

Statistical analyses of experimental results were performed with one-way ANOVAs with the Bonferroni correction. A *p*-value of <0.05 and <0.01 was considered statistically significant. Data are presented as the mean ± SEM. All experiments were performed at least three times.

## Data Availability

Not applicable.

## Supplementary Materials

The supporting information can be downloaded at: https://www.mdpi.com/article/10.3390/ijms231911636/s1.
