# Supplementary material for: Evogliptin Directly Inhibits Inflammatory and Fibrotic Signaling in Isolated Liver Cells
Source: Int J Mol Sci. 2022 Oct 1;23(19):11636. doi: 10.3390/ijms231911636 (PMC9569597; doi:10.3390/ijms231911636)
Supplement: Supplementary file 1 [file ijms-23-11636-s001.zip › ijms-1909702-supplementary.pdf]

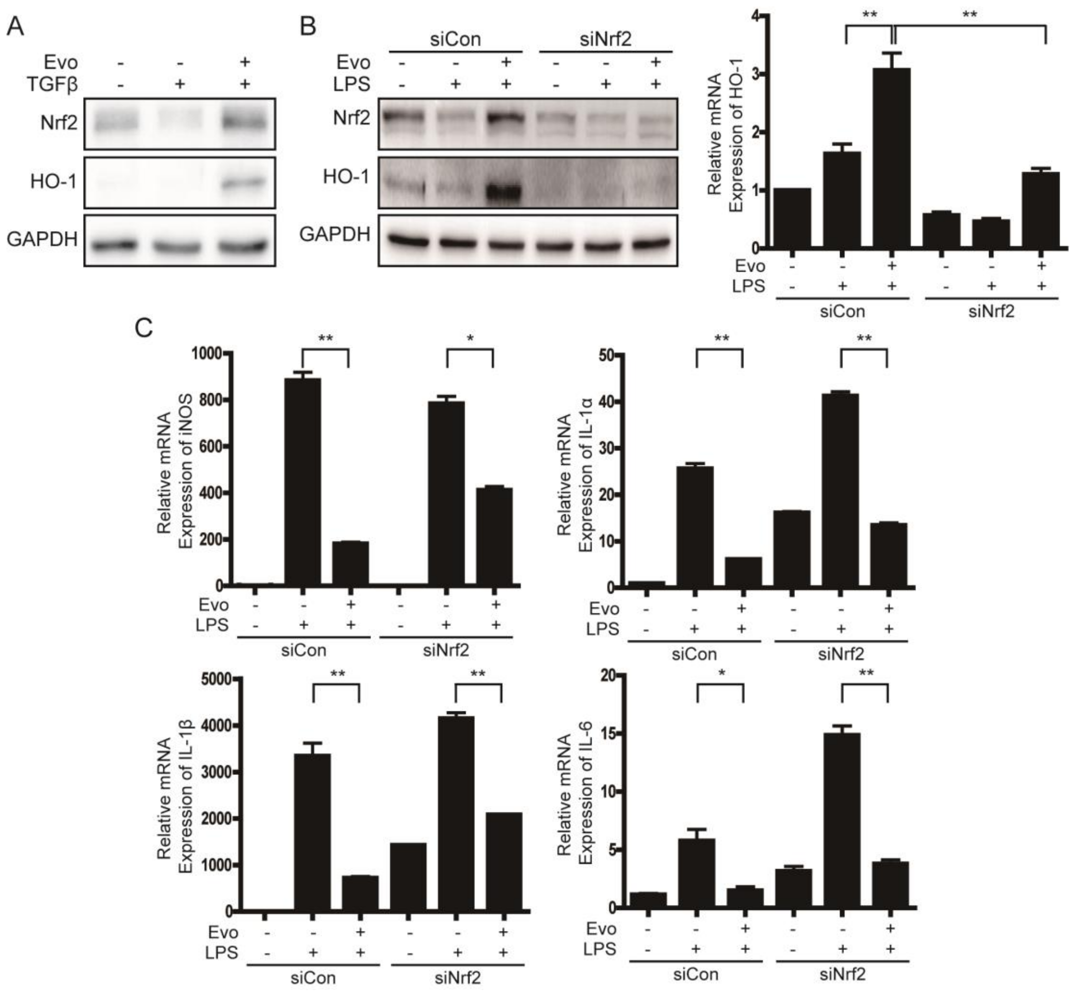

Figure S1. The effect of evogliptin on Nrf2/HO-1 expression.

(**A**) Primary hepatocyte was pretreated with LPS for 2h, and then exposed to evogliptin for 24 h. The expression of Nrf2 and HO-1 analyzed by western blot. Data represented in the bar graph are the mean ± SEM of three independent measurements. **P < 0.01. (B) Primary HC transfected with Nrf2 siRNA or control (Con)-siRNA for 48 h were subjected to western blot analysis and real-time RT-PCR analysis. (C) Primary HC transfected with Nrf2 siRNA or control (Con)-siRNA for 48 h were subjected to real-time RT-PCR analysis using primers specific for Nrf2, HO-1, iNOS, IL1α, IL1β and IL6. *P < 0.05, **P < 0.01.

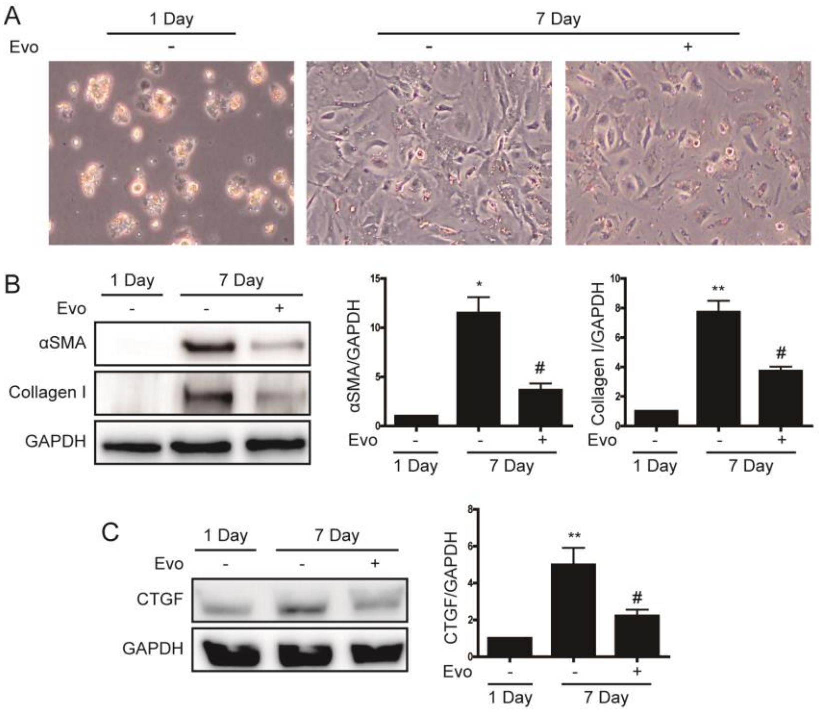

Figure S2. The inhibitory effect of evogliptin on hepatic stellate cell activation.

(**A**) Primary hepatic stellate cells morphology was analyzed using microscopy. Primary HSCs were cultured for 2 h, after which unattached cells and debris were removed by washing. HSCs were further cultured for three, five, and 7 days in DMEM containing 0.5% FBS with or without evogliptin. (**B,C**) Western blot analysis of SMA, collagen and CTGF expression in cultured HSCs. Data in the graph are represented as the mean ± SEM. *P<0.05, **P<0.01 relative to the 1 day. #P<0.05 relative to the 7 days.
